# A Broadband Differential-Fed Dual-Polarized Hollow Cylindrical Dielectric Resonator Antenna for 5G Communications

**DOI:** 10.3390/s20226448

**Published:** 2020-11-11

**Authors:** Xiaosheng Fang, Kangping Shi, Yuxiang Sun

**Affiliations:** 1 Department of Electronic and Information Engineering, Shantou University, Shantou 515000, China; 19kpshi@stu.edu.cn; 2 Guangdong Provincial Key Laboratory of Digital Signal and Image Processing, Department of Electronic and Information Engineering, Shantou University, Shantou 515000, China; 3 College of Electronics and Information Engineering, Shenzhen University, Shenzhen 518000, China; yxsun4-c@my.cityu.edu.hk

**Keywords:** differential-fed, dual-polarized, hollow cylindrical DRA, broadband

## Abstract

A broadband differential-fed dual-polarized hollow cylindrical dielectric resonator antenna (DRA) is proposed in this article. It makes use of the HEM_111_, HEM_113_, and HEM_115_ modes of the cylindrical hollow DRA. The proposed DRA is simply fed by two pairs of conducting strips and each pair of strips is provided with the out-of-phase signals. After introducing four disconnected air holes into the DRA, a broadband characteristic is achieved, with little effect on the antenna gain of its higher-order modes. To verify this idea, frosted K9-glass is applied to fabricate the hollow cylindrical DRA. The differential S-parameters, radiation patterns, and antenna gain of the DRA are studied. It is found that the proposed differential-fed dual-polarized DRA is able to provide a broad differential impedance bandwidth of ~68% and a high differential-port isolation better than ~46 dB. Moreover, symmetrical broadside radiation patterns are observed across the whole operating band. The proposed DRA covers the frequency bands including the 5G-n77 (3.4–4.2 GHz), 5G-n79 (4.4–5.0 GHz), WLAN-5.2 GHz (5.15–5.35 GHz), and WLAN-5.8 GHz (5.725–5.825 GHz), which can be used for 5G communications.

## 1. Introduction

The dielectric resonator antenna (DRA) [1,2,3] has been widely investigated in the past three decades. It has lots of attractive characteristic such as light weight, ease of excitation, and being bandwidth-controllable. In recent years, the dual-polarized antenna has received more and more attention. Its main advantage is to save the number of antennas in the base-station system. In addition, it can improve the channel capacity and reduce co-channel interference. As a result, many studies on the dual-polarized DRA have been reported [4,5,6,7,8,9,10]. For example, in [4], a dual-polarized DRA fed by a coplanar waveguide is proposed. In this design, even and odd modes of the coplanar waveguide structure are utilized, with a relatively narrow impedance bandwidth of ~7% obtained. The bandwidth of the dual-polarized DRA can be enlarged by using suitable excitation. For instance, in [5], two pairs of balanced probes are used to excite the two orthogonal mode of the HEM_111_ mode of the cylindrical DRA, with an impedance bandwidth of ~20% obtained. In addition, the dual-mode method can be used to widen the bandwidth. For instance, in [6], a dual-polarized cylindrical DRA is designed using its HEM_111_ and HEM_113_ modes, with an impedance bandwidth of ~30% reported. The disadvantage is that the feeding method of the two ports is not consistent, leading to the different and asymmetric radiation patterns [6].

On the other hand, because of the high-level integration with other solid-state circuits, the on-chip DRA [11,12,13] can meet the requirements of small size and low cost in high-frequency system. However, for the chip-sets in silicon technology, traditional singly-fed antennas are easily affected by the substrate noise and power supply variations. A differential-fed antenna can avoid this problem because it allows lower offset and higher linearity [14,15]. Hence, differential-fed DRA [16,17,18,19,20,21,22,23,24,25,26] with excellent performance has great potential for high frequency on-chip communication applications. Recently, some efforts have been devoted to the design of the differential dual-polarized DRAs [27,28,29,30,31]. For example, the differential-fed dual-polarized DRAs with dual band performance [27] or filtering characteristic [28] have been reported. The works [27,28] show that using the differential feeding can suppress the unwanted DRA modes. Moreover, it has an important merit that the isolation between the two differential ports is much higher than that of the single-ended version.

The hollow DRAs [32,33,34,35,36,37] have been extensively studied for the merit of wide impedance bandwidth, but at the cost of high cross-polarization. This can be improved by using the differential-fed method since it can cancel out the leakage radiation. However, relatively few studies have been done on broadening the differential impedance bandwidth of the DRA (calculated from the mixed-mode S parameters S_dd11_).

In this paper, a broadband differential-fed dual-polarized cylindrical DRA is investigated. With the introduction of four disconnected air holes in the DRA, three designated DRA modes (HEM_111_, HEM_113_, and HEM_115_ modes) merge together and form a broadband dual-polarized DRA. Most importantly, the antenna gain of the higher-order modes is maintained. The results show that the proposed DRA can provide a broad differential impedance bandwidth of ~68% for each differential-port, with a good differential port-isolation higher than ~46 dB achieved.

The rest of this paper will be organized as follows. In Section 2, the theory of the differential-fed dual-polarized antenna is discussed. In Section 3, the configuration of the proposed DRA is given. Additionally, the effect of the air region on the performance of the DRA is studied. In Section 4, the performance of the proposed DRA is shown and assessed. Finally, a conclusion is given in Section 5.

## 2. Theory

### 2.1. S Parameters of the Dual-Polarized Antennas Using the Single-Ended and Differential Ports

In designing the dual-polarized antennas, the port matching and port isolation are needed to considered. Table 1 compares the S parameters of the dual-polarized antenna with two single-ended ports and differential ports. When the ports are single-ended, the port matching (S_11_ and S_22_) and port isolation (S_12_ and S_21_) are considered. However, it is different as the ports are differential ones. In this condition, the port matching is defined as S_dd11_ and S_dd22_, while the port isolation is defined as S_dd12_ and S_dd21_. In [38,39,40], a dual-polarized antenna with two differential-ports is considered as a single-ended four-ports network. Its differential S parameters is given as:

$$S_{dd11}=(S_{11}-S_{12}-S_{21}+S_{22})/2$$

$$S_{dd22}=(S_{33}-S_{34}-S_{43}+S_{44})/2$$

$$S_{dd21}=(S_{31}-S_{41}-S_{32}+S_{42})/2$$

$$S_{dd12}=(S_{13}-S_{14}-S_{23}+S_{24})/2$$

where S*_ij_* (*i* = 1, 2, 3, 4; *j* = 1, 2, 3, 4) denotes the single-ended S-parameters. As can be seen from the above formulas, the matching of the differential ports (S_dd11_ and S_dd22_) are related to the matching (S_11_ and S_22_) and isolation (S_12_ and S_21_) of the single-ended ports.

### 2.2. Strips-Fed Dual-Polarized Cylindrical DRA Using Single-Ended and Differential Ports

**Figure 1:**
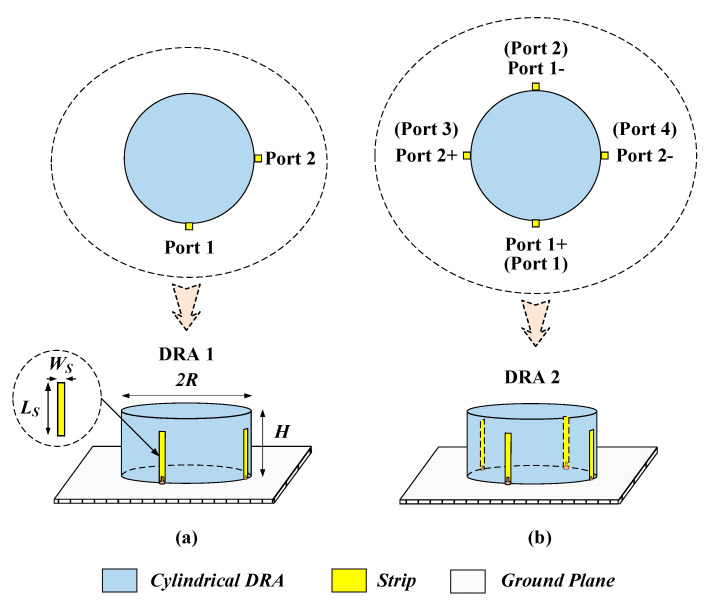
Configuration of the strips-fed dual-polarized cylindrical DRAs. (**a**) Two single-ended ports; (**b**) Two differential ports.

**Figure 2:**
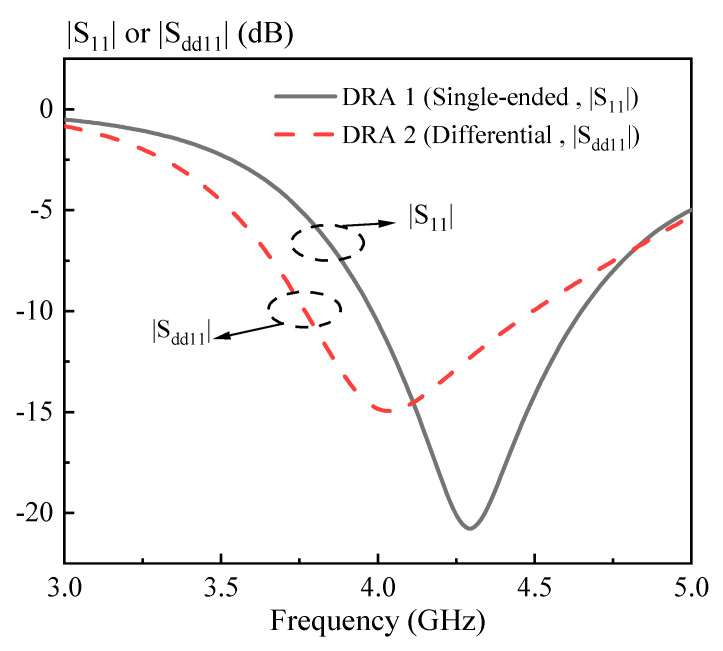
Reflection coefficient of the strips-fed dual-polarized cylindrical DRAs shown in Figure 1. The |S_11_| is for DRA 1 (single-ended port) and |S_dd11_| is for DRA 2 (differential port).

**Figure 3:**
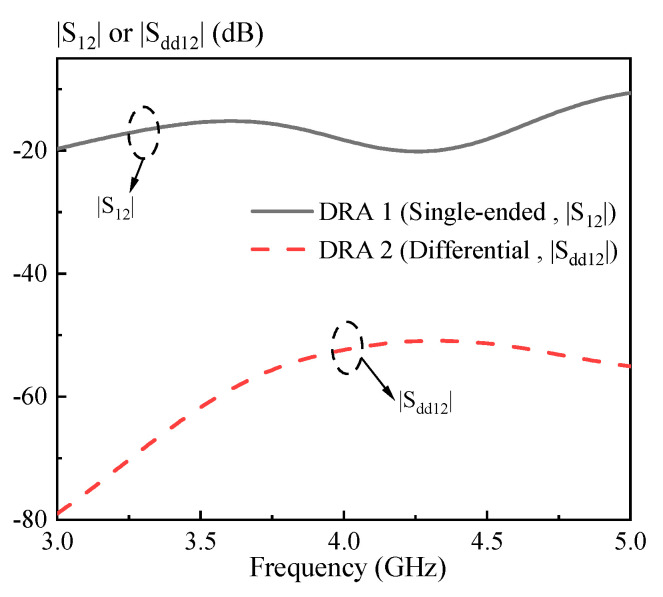
Port isolation of the strips-fed dual-polarized cylindrical DRAs shown in Figure 1. The |S_12_| is for DRA 1(single-ended port) and |S_dd12_| is for DRA 2 (differential port).

In this section, a strips-fed cylindrical DRA is studied to compare the performance of the dual-polarized antennas using the single-ended and differential ports. Figure 1 shows the structure of the DRAs, where Figure 1a uses two single-ended ports and Figure 1b employs two differential ports. The cylindrical DRA (*εr* = 6.85) has a radius of *R* = 12 mm and a height of *H* = 10 mm. For each port, a strip having a width of *W_s_* = 1 mm and a length of *L_s_* = 9 mm is adhered to the sidewall of the cylindrical DRA, which is used to excite its fundamental HEM_111_ mode. Figure 2 shows the port matching for both cases. Referring to the figure, the 10dB-impedance bandwidth of the single-ended (|S_11_| ≤ −10dB) and differential (|S_dd11_| ≤ −10dB) versions are obtained as ~16% (3.98–4.65 GHz) and ~17% (3.77–4.49 GHz), respectively. Figure 3 shows the port isolation for both cases. It can be seen from the figure that the single-ended port isolation is only higher than 16 dB (3.98–4.65 GHz), while the port isolation of the differential version is higher than 45 dB (3.77–4.49 GHz). This shows the isolation of the differential version is better than that of the single-ended. The reason is given here. For the differential version (Figure 1b), ports 3 and 4 are symmetrical about the plane of ports 1 and 2. Under this condition, S_13_ = S_14_ and S_23_ = S_24_ are obtained, resulting in S_dd12_ = 0. In other words, the isolation of the differential version is infinite in theory.

## 3. Antenna Configuration, Air Region Effect and Resonant Modes

### 3.1. Antenna Configuration

**Figure 4:**
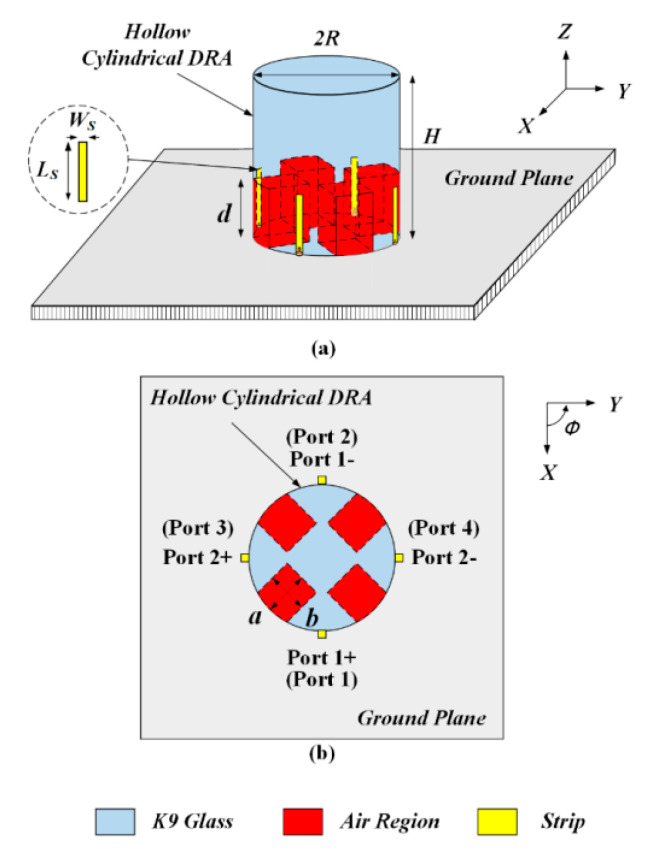
The configuration of the proposed broadband differential-fed dual-polarized hollow cylindrical DRA: *a* = 6 mm, *b* = 7 mm, *d* = 9 mm, *R* = 11 mm, *H* = 25 mm, *L*_S_ = 9 mm, *W*_S_ = 1 mm, and *ε*_r_ = 6.85. (**a**) Perspective view. (**b**) Top view.

Figure 4 shows the configuration of the proposed broadband differential-fed dual-polarized hollow cylindrical DRA locating on a ground plane of 12 × 12 cm^2^. The hollow cylindrical DRA made of frosted K9 glass (*ε_r_* = 6.85) has a radius of *R* = 11 mm and a height of *H* = 25 mm (*H*/*R* = 2.27). It should be mentioned that the value of *H/R* cannot be too small, otherwise the two higher-order modes (HEM_11__3_ and HEM_11__5_ modes) would be far away from the fundamental mode (HEM_111_ mode) [41] and they will not be able to merge. In our experience, *H/R* should be larger than 2. Four approximate rectangular air holes are dug at the bottom of the DRA. Each air hole has a length of *a* = 6 mm, a width of *b* = 7 mm, and a height of *d* = 9 mm. Four metal strips were employed to excite the hollow cylindrical DRA, which has a length of *Ls* = 9 mm and a width of *Ws* = 1 mm. These strips are connected with four single-ended ports (1, 2, 3, 4), which form two differential ports (1+, 1−) and (2+, 2−), respectively.

### 3.2. The Effect of the Air Region

**Figure 5:**
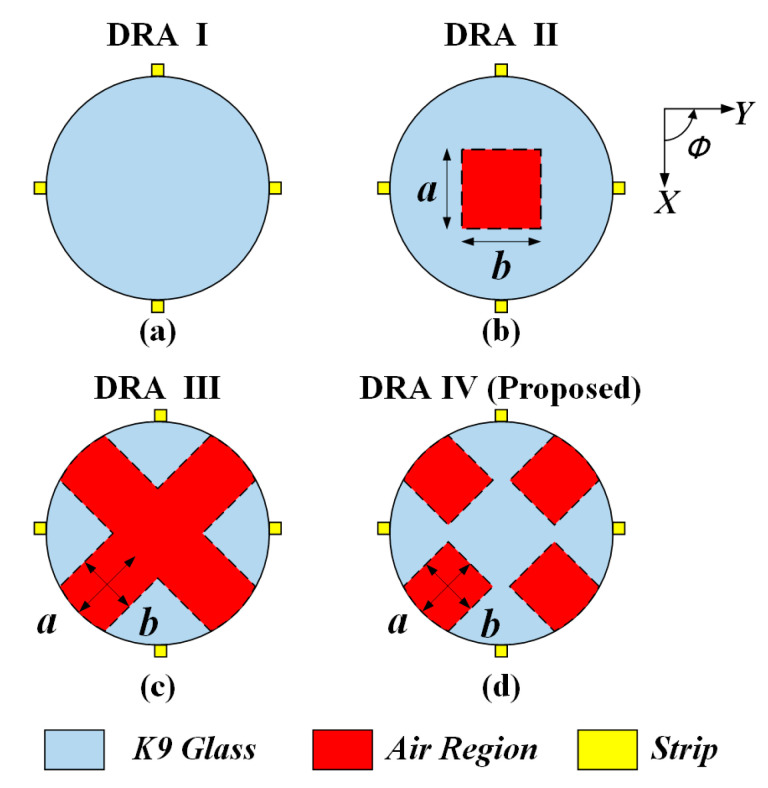
Top view of four differential-fed dual-polarized DRAs. (**a**) Solid cylindrical DRA; (**b**) Hollow cylindrical DRA with a rectangular air hole (*a* = 8 mm, *b* = 8 mm) at its center; (**c**) Hollow cylindrical DRA with four connected approximate rectangular air holes (*a* = 6 mm, *b* = 8 mm); (**d**) Hollow cylindrical DRA with four disconnected approximate rectangular air holes (*a* = 6 mm, *b* = 7 mm). All the air holes are at the bottom and have the same height *d =* 9 mm.

**Figure 6:**
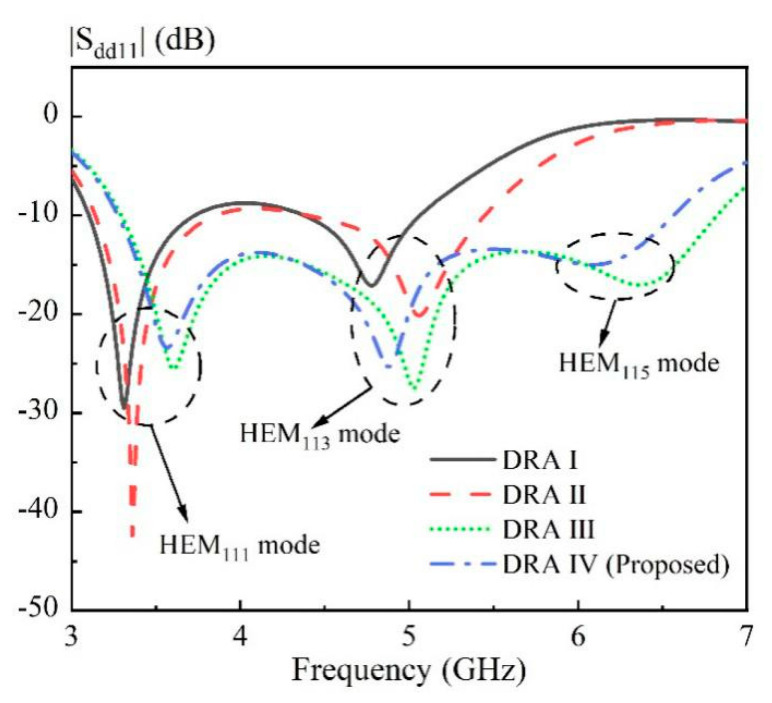
Comparison of the simulated |S_dd11_| of four DRAs shown in Figure 5.

**Figure 7:**
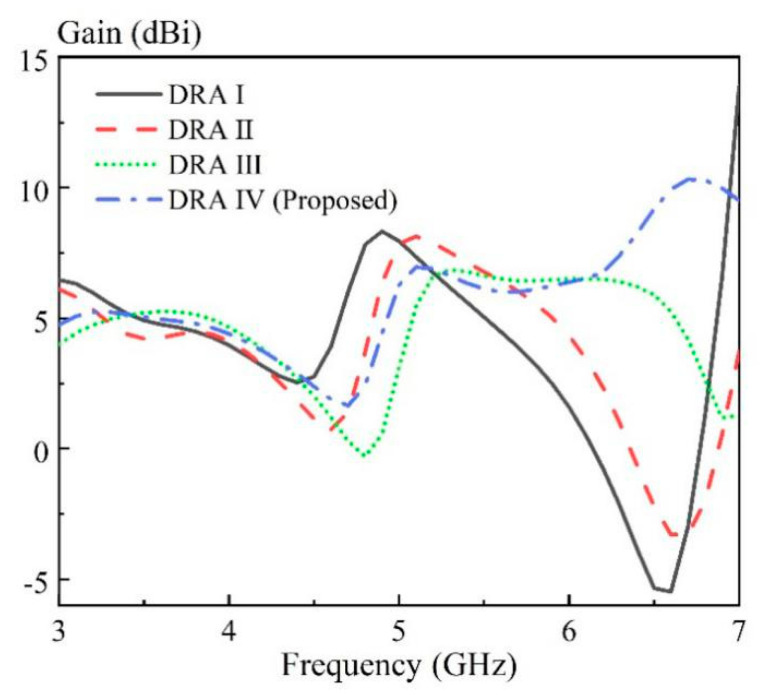
Comparison of the simulated antenna gain at differential-port 1 of four DRAs shown in Figure 5.

**Figure 8:**
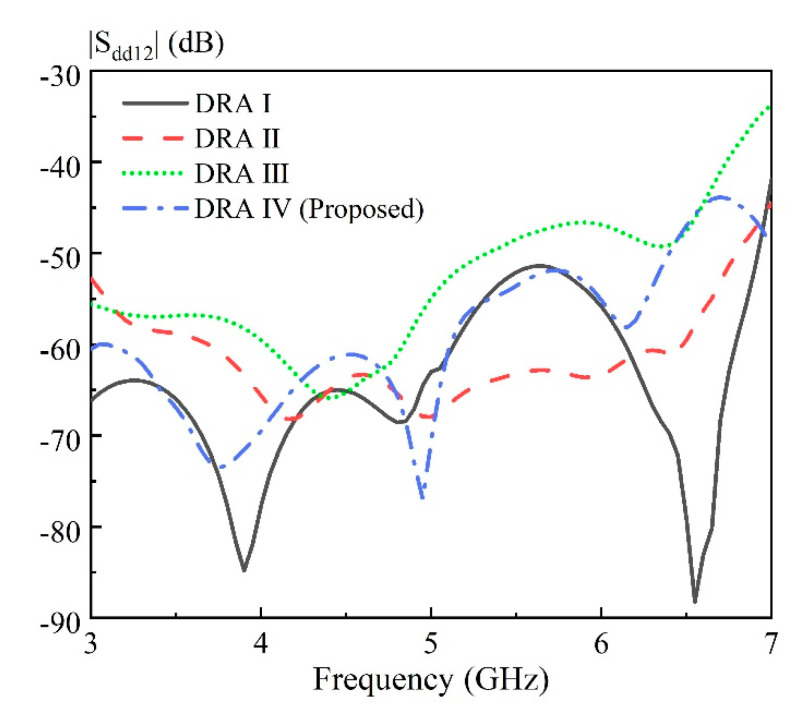
Comparison of the simulated |S_dd12_| of four DRAs shown in Figure 5.

The effect of the air region on the antenna performance is discussed here. Figure 5 shows the solid cylindrical DRA (Figure 5a), as well as three kinds of hollow DRAs (Figure 5b–d) with different air region distributions. The excitation method and ground plane used in Figure 5 is the same as that in Figure 4. For brevity, the ground plane is not shown here. The corresponding simulated |S_dd11_| at differential-port 1 is shown in Figure 6. It should be mentioned that the definition of the differential S parameters for a differential-fed dual-polarized antenna has been given in Table 1. With reference to the result of the |S_dd11_| for DRA I, the HEM_111_ and HEM_113_ mode of the cylindrical DRA are excited at 3.31 and 4.78 GHz, respectively. However, its matching around 4.0 GHz is not very good, which makes it impossible to achieve a wideband antenna. A similar result of |S_dd11_| is observed for DRA II having a rectangular hollow region at its center. To further enhance the bandwidth, DRA III and DRA IV with four approximate rectangular air holes are designed, in which the air holes are connected in DRA III and disconnected in DRA IV. With reference to the results of |S_dd11_| for DRA III and DRA IV, the HEM_111_, HEM_113_ and HEM_115_ modes of the DRA are successfully excited to form a broadband DRA. This is reasonable because the insertion of the air region inside the DRA reduces its effective dielectric constant, and therefore enhances overall bandwidth. Furthermore, as can be seen from Figure 7, the antenna gain of the DRA III ranging from 6.5 to 7.0 GHz has a sharp decline; this is because the hollow region at the center destroys the first standing wave of the HEM_115_ mode more seriously. The above discussion implies that the total volume of the hollow region should be large enough so that the differential impedance bandwidth can be obviously increased. However, the hollow region should be avoided in the middle of the DRA, so as to minimize the influence on the antenna gain of the higher-order HEM_115_ mode. In addition, it should be mentioned that good isolation |S_dd12_| between the two differential-ports is obtained for the four cases, with their results shown in Figure 8.

### 3.3. Resonant Modes

**Figure 9:**
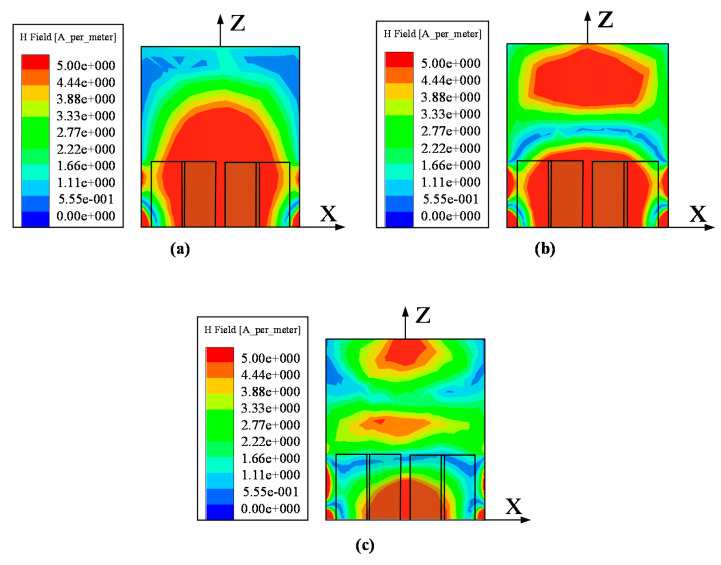
Simulated H-fields (x–z plane) inside the proposed differential-fed dual-polarized DRA. (**a**) HEM_111_^y^ mode at 3.56 GHz. (**b**) HEM_113_^y^ mode at 4.88 GHz. (**c**) HEM_115_^y^ mode at 6.08 GHz.

The resonant modes of the proposed hollow cylindrical DRA (DRA IV) are verified by the corresponding near field in this section. The H-fields (x–z plane) of the proposed DRA at the resonance frequencies are given in Figure 9. The analysis is similar with that in [42]. With reference to Figure 9a, the typical H-field of the HEM_111_^y^ mode was found at 3.56 GHz, which has one energy concentration zone inside the DRA. Referring to Figure 9b,c, two and three energy concentration zones along z-direction were observed, respectively. This denotes the resonant modes at 4.88 GHz and 6.08 GHz are caused by the HEM_113_^y^ and HEM_115_^y^ modes, respectively.

### 3.4. Parametric Study

**Figure 10:**
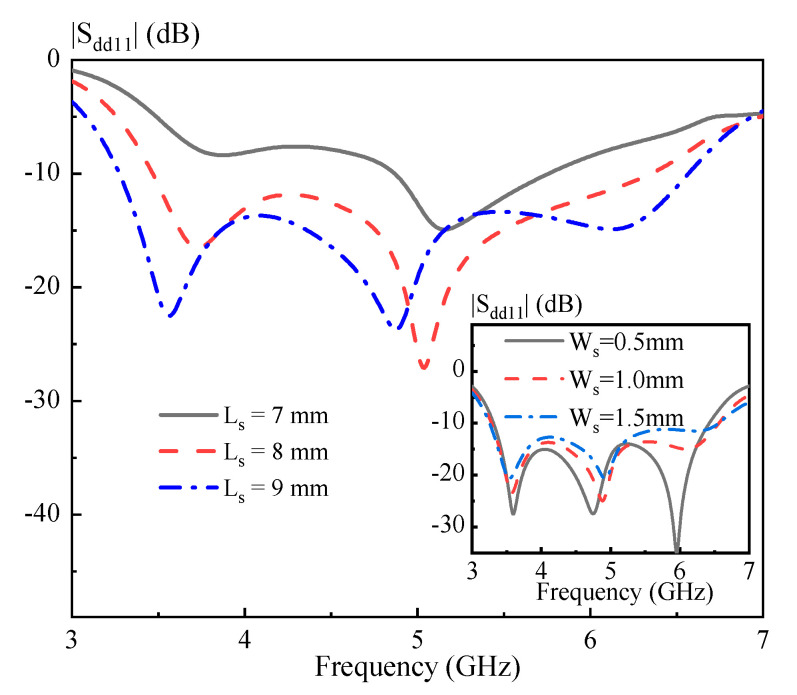
Simulated |S_dd11_| of the proposed DRA versus frequency for different *L_s_*. The inset shows the simulated |S_dd11_| of the proposed DRA versus frequency for different *W_s_*.

**Figure 11:**
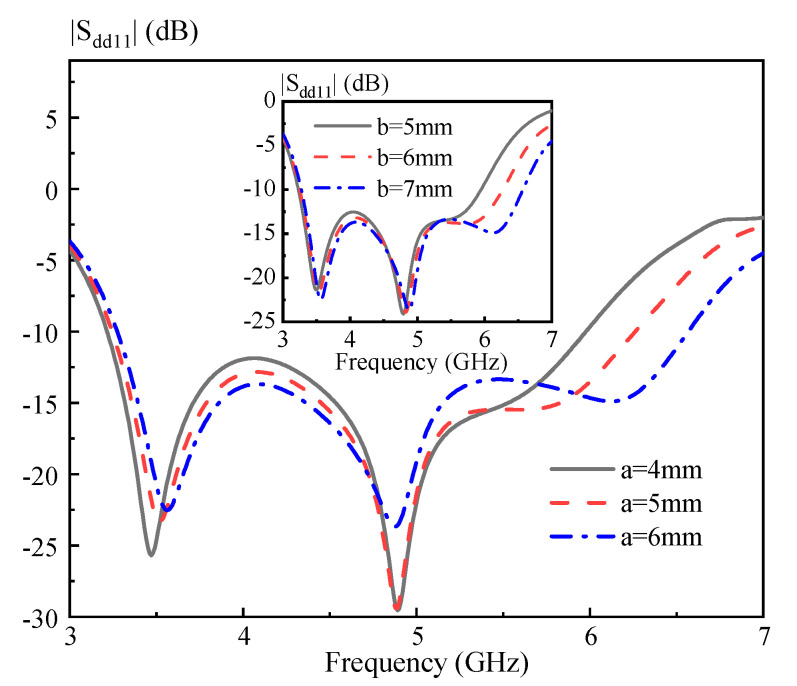
Simulated |S_dd11_| of the proposed DRA versus frequency for different *a*. The inset shows the simulated |S_dd11_| of the proposed DRA versus frequency for different *b*.

**Figure 12:**
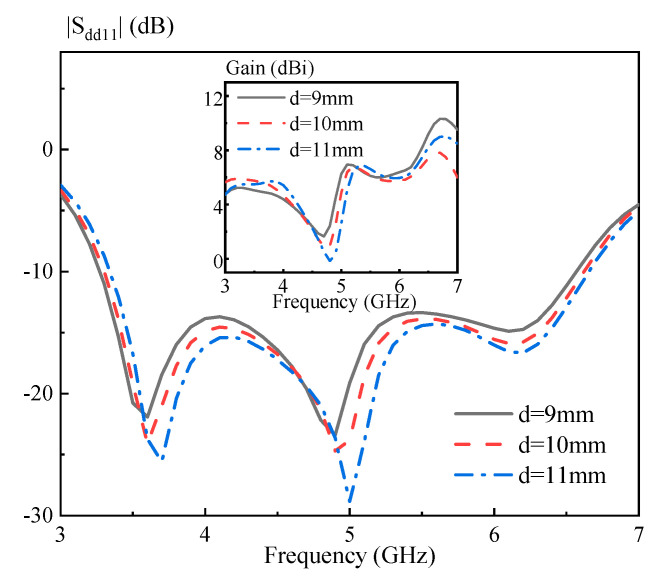
Simulated |S_dd11_| of the proposed DRA versus frequency for different *d*. The inset shows the simulated antenna gain of the proposed DRA at the differential-port 1 versus frequency for different *d*.

In this part, parametric study is carried out to characterize the proposed DRA. Figure 10 shows the simulated |S_dd11_| versus frequency with the length of the strips *L_s_* = 7, 8, and 9 mm. Referring to the figure, increasing *L_s_* would improve the matching of the DRA, and a good result is obtained as *L_s_* = 9 mm. Its inset gives the simulated |S_dd11_| versus frequency with the width of the strips *W_s_* = 0.5, 1, and 1.5 mm. As can be seen from the figure, altering *W_s_* has little effect on the matching of the DRA. Next, the sizes of the hollow region on the effect of the DRA performance is investigated. Figure 11 shows the simulated |S_dd11_| versus frequency with the length of the hollow region *a* = 4, 5, and 6 mm. Its inset shows the simulated |S_dd11_| versus frequency with the width of the hollow region *b* = 5, 6, and 7 mm. With reference to the figure, increasing *a* and *b* would shift the third resonance modes obviously upward, and thus enhance the differential impedance bandwidth of the DRA. Figure 12 presents the simulated |S_dd11_| of the proposed DRA versus frequency for different *d* = 8, 9, and 10 mm. Referring to the figure, changing *d* has a small effect on the differential impedance bandwidth of the DRA. However, a larger *d* would have a negative effect on the antenna gain around 6.5 GHz. Based on the above analysis, a design guideline is also concluded as follows:

(1) Determining the dimension of the cylindrical DRA: *R* ~ 0.47 *λ_d_* and *H* ~ 1.07 *λ_d_,* in which *λ_d_* is the wavelength in the dielectric corresponding to the center frequency;
(2) Introducing four rectangular hollow regions (*a* ~ 0.26 *λ_d_*, *b* ~ 0.3 *λ_d_* and *d* ~ 0.39 *λ_d_*) into the DRA, and then adjusting *L_s_* to obtain a good matching;
(3) Slightly adjusting *a* and *b* to obtain an optimal differential impedance bandwidth of the DRA;
(4) Slightly adjusting *d* to optimize the antenna gain of the high frequency band.

## 4. Results

**Figure 13:**
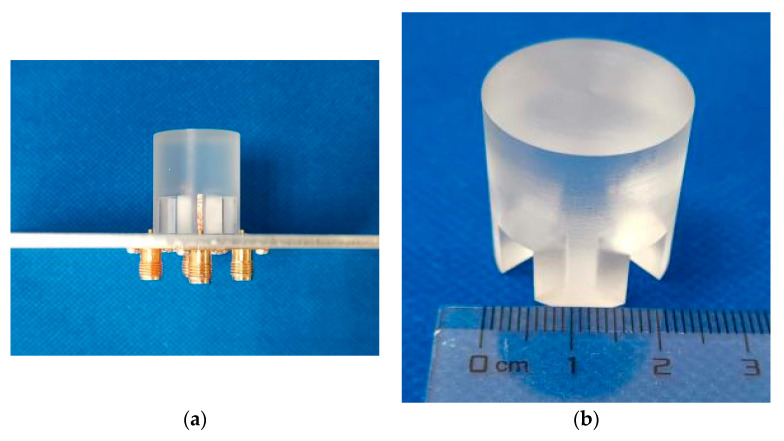
Prototype of the proposed broadband differential-fed dual-polarized hollow cylindrical DRA. (**a**) Overview of the antenna. (**b**) The hollow cylindrical DRA fabricated using frosted K9 glass.

**Figure 14:**
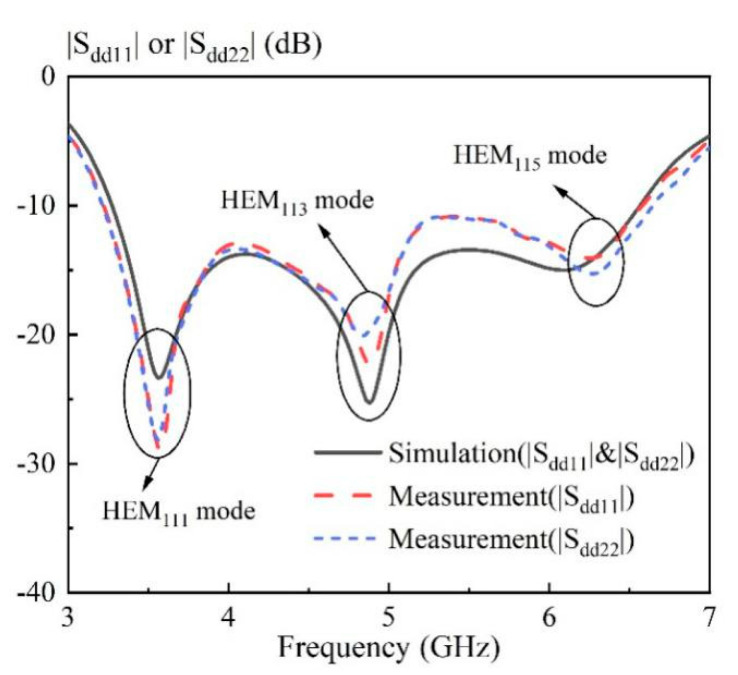
Simulated and measured |S_dd11_| and |S_dd22_| of the proposed DRA versus frequency.

**Figure 15:**
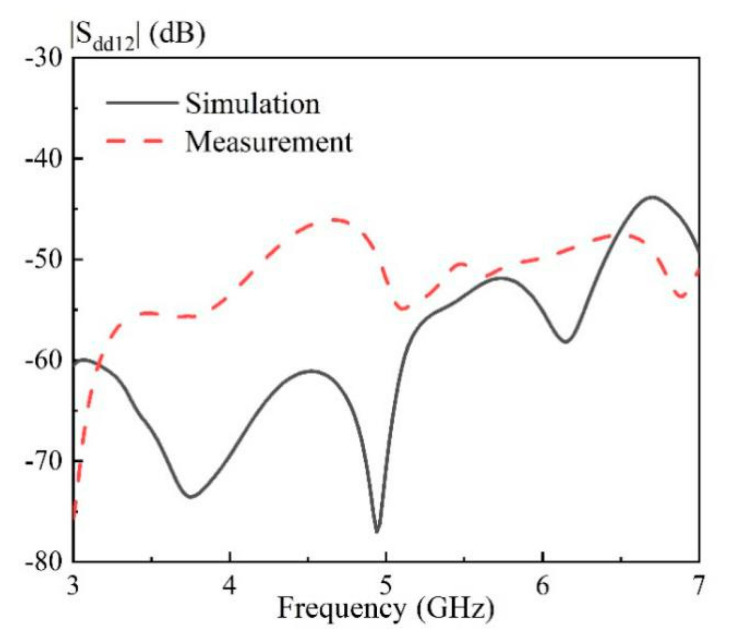
Simulated and measured |S_dd12_| of the proposed DRA versus frequency.

In this part, the measured results of the proposed broadband differential-fed dual-polarized hollow cylindrical DRA are reported. The DRA was fabricated using frosted K9 glass, with its prototype shown in Figure 13. Figure 14 shows the measured and simulated differential S-parameters of the proposed DRA at two differential-ports. With reference to the figure, the three resonance frequencies for the differential-port 1 are measured as 3.58 GHz, 4.88 GHz and 6.26 GHz, respectively. This agrees with the simulated resonance frequencies of 3.56 GHz (0.55% error), 4.88 GHz (0.00% error), and 6.08 GHz (2.87% error). A similar result was obtained for the differential-port 2. The measured differential impedance bandwidth (|S_dd11_|&|S_dd22_| ≤ −10 dB) at the differential-port 1 and 2 are obtained as 68.4% (3.23–6.59 GHz) and 69.4% (3.22–6.64 GHz), respectively. The above results are summarized in Table 2 for the ease of reference. Figure 15 shows the measured and simulated isolation between two differential-ports. As can be seen from the figure, the isolation between differential-port 1 and 2 (|S_dd12_|) is less than 46 dB across the whole operating band.

**Figure 16:**
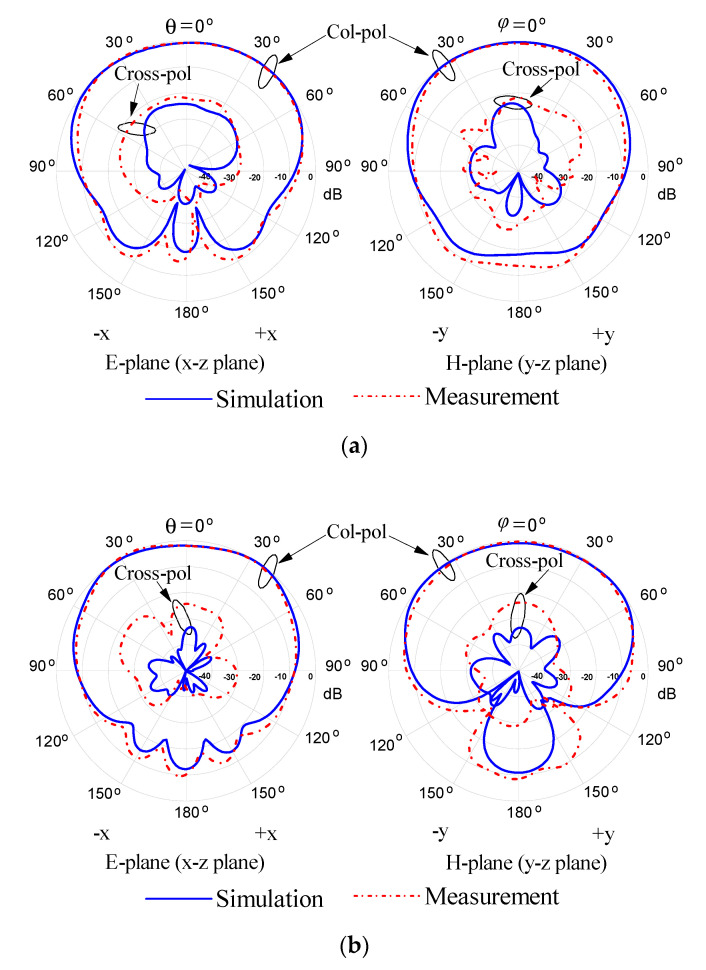

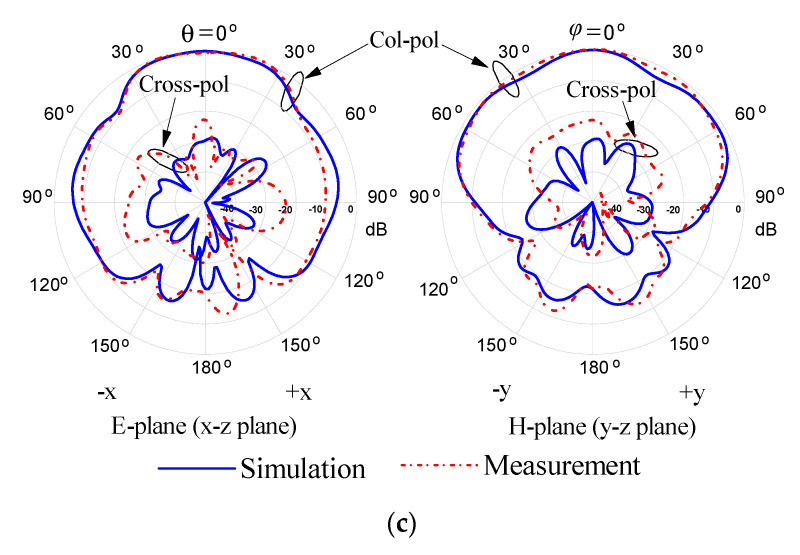
Simulated and measured radiation patterns of the proposed DRA at differential-port 1. (**a**) 3.56 GHz. (**b**) 4.88 GHz. (**c**) 6.08 GHz. 
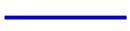
 Simulation 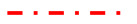 Measurement.

The simulated and measured radiation patterns of the proposed DRA at differential-port 1 for the three DRA modes are shown in Figure 16. In the measurement, a broadband external 180° hybrid coupler (2–18 GHz) was applied to provide the differential signals. Referring to the figure, symmetrical broadside radiation patterns were presented for the three resonant modes. In the boresight direction, the co-polarized field is stronger than its cross-polarized counterpart by more than 20 dB for the E- and H-planes. It is also observed that the level of the cross-polarization field of all angles is smaller than −20 dB, which is due to the use of differential feeding.

**Figure 17:**
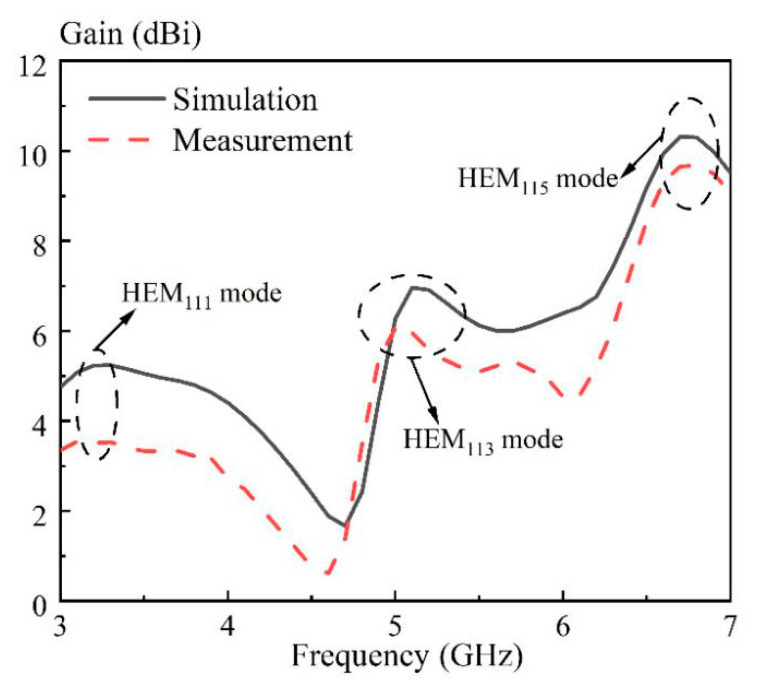
Simulated and measured antenna gain of the proposed DRA at differential-port 1 versus frequency.

**Figure 18:**
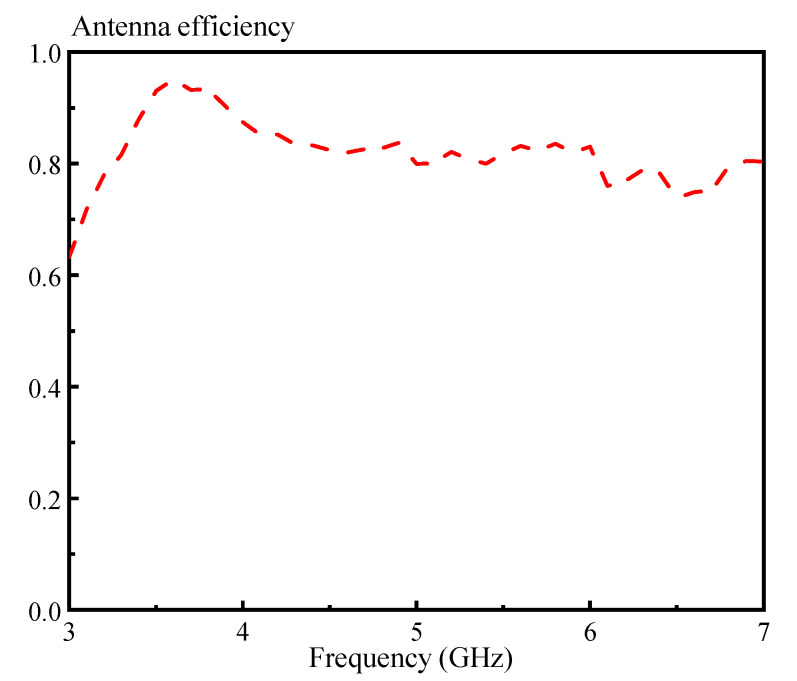
Measured antenna efficiency of the proposed DRA at differential-port 1 versus frequency.

**Figure 19:**
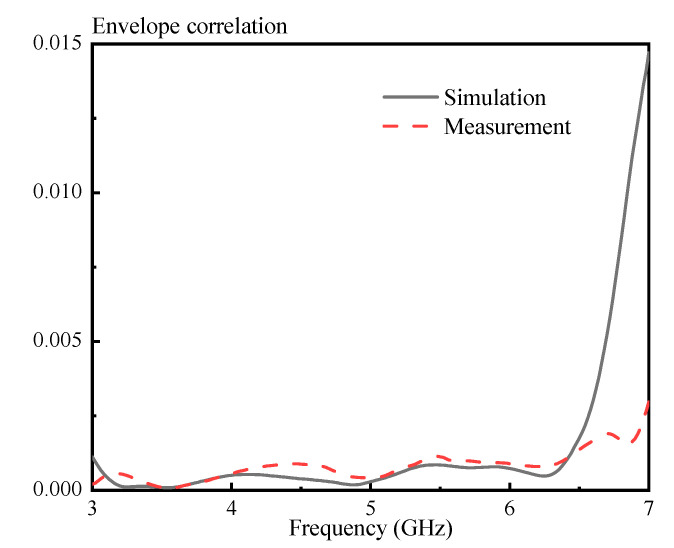
Simulated and measured envelope correlations of the proposed DRA.

Figure 17 presents the simulated and measured antenna gains at the differential-port 1. Referring to the figure, the three measured peak gains are obtained as 3.54 dBi (@3.1 GHz), 6.06 dBi (@5.0 GHz) and 9.67 dBi (@6.8 GHz), which are due to the HEM_111_, HEM_113_, and HEM_115_ modes, respectively. It is found that the peak gain of the three DRA modes increase in turn, showing that the air holes have a relatively small effect on the antenna gain of the higher-order modes. The measured antenna efficiency of the proposed DRA at the differential-port 1 is also given in Figure 18. With reference to the figure, its range is from 0.74 to 0.95 in the usable frequency band (3.23–6.59 GHz). The radiation patterns, antenna gain and efficiency at the differential-port 2 are very similar with that of the differential-port 1. For brevity, the results are not given here. Figure 19 shows the simulated and measured envelope correlations (ECs) of the proposed DRA. The formula provided in [43] is used to calculate this parameter. With reference to the figure, both the simulated and measured ECs are smaller than 0.005 across the whole frequency band, satisfying the requirement of the MIMO system (EC ≤ 0.5).

Finally, the performance of the proposed differential-fed dual-polarized DRA is assessed. Table 3 summarizes the different dual-polarized DRAs. As can be seen from the table, using the differential feeding ([27] and proposed) can obtain a better isolation than the single-ended feeding. In addition, compared with other works [4,5,6,7,8,27], our DRA has a broader impedance bandwidth and higher antenna peak gain, with an excellent isolation and medium cross-polarization level obtained. Additionally, its consistence of the radiation patterns observed at two ports is good. However, the cost is that a DRA of a relatively large size is required.

## 5. Conclusions

A broadband differential-fed dual-polarized hollow cylindrical DRA has been investigated. The HEM_111_, HEM_113_, and HEM_115_ modes of the hollow cylindrical DRA have been used for the design. The effect of the air region distribution on the performance of the hollow DRA has been investigated. It has been found that the hollow DRA with four disconnected air holes can have a broad differential impedance bandwidth and good antenna gain. It shows that the proposed DRA has a measured differential impedance bandwidth of ~68% for each differential-port. Furthermore, a good differential-port isolation higher than ~46 dB has been obtained. The proposed DRA covers the frequency bands including the 5G-n77, 5G-n79, WLAN-5.2 GHz, and WLAN-5.8 GHz. Additionally, the proposed design concept is potentially suitable for designing the on-chip DRA at high frequency.

## Figures and Tables

**Table 1:** Comparison of the Dual-Polarized Antennas with Two Single-ended Ports and Differential Ports.

Port Distribution	S Parameter	Diagrammatic Sketch
Two single-ended ports	Port matching: S_11_, S_22_ Port isolation: S_21_, S_12_	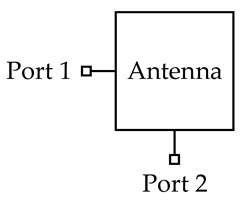
Two differential ports	Port matching: $S_{dd11}=\left(S_{11}-S_{12}-S_{21}+S_{22}\right)/2$ $S_{dd22}=\left(S_{33}-S_{34}-S_{43}+S_{44}\right)/2$	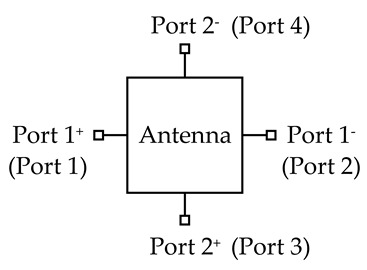
	Port isolation: $S_{dd21}=\left(S_{31}-S_{41}-S_{32}+S_{42}\right)/2$ $S_{dd12}=\left(S_{13}-S_{14}-S_{23}+S_{24}\right)/2$	

**Table 2:** Summary of the resonance frequency and impedance bandwidth of the proposed differential-fed dual-polarized DRA.

Diff. Port (*i*)	Resonant Modes	Resonance Frequency			Mea. Diff. Impedance Bandwidth
		Sim. (GHz)	Mea. (GHz)	Error (%)	
1	HEM_111_^y^	3.56	3.58	0.55	68.4% (3.23–6.59 GHz)
	HEM_113_^y^	4.88	4.88	0.00	
	HEM_115_^y^	6.08	6.26	2.87	
2	HEM_111_^x^	3.56	3.56	0.00	69.4% (3.22–6.64 GHz)
	HEM_113_^x^	4.88	4.84	0.83	
	HEM_115_^x^	6.08	6.26	2.87	

**Table 3:** Comparison of the Different Dual-Polarized DRAs.

	Size (*λ_d_^3^*)	Impedance Bandwidth (%)	Isolation (dB) (Worst within Band)	X-pol Level (dB)	Peak Gain (dBi)	Feeding Type	Consistence of the Radiation Patterns at Two Ports
Ref. [4]	0.219	7	25	−20	6.45	single-ended	Medium
Ref. [5]	0.472	20	25	−30	NA.	single-ended	Good
Ref. [6]	0.826	31.7	37	−20	9.42	single-ended	Medium
Ref. [7]	0.294	7.9	36	−34	6.8	single-ended	Medium
Ref. [8]	0.25	25	20	−15	7.4	single-ended	Good
Ref. [27]	0.580	4.3	47	−33	6.94	Differential	Good
Proposed	0.747	68	46	−22	9.67	Differential	Good

## References

[1] Long S.A., McAllister M.W., Shen L.C. (1983). The resonant cylindrical dielectric cavity antenna. IEEE Trans. Antennas Propag..

[2] Luk K.M., Leung K.W. (2003). Dielectric Resonator Antennas.

[3] Petosa A. (2007). Dielectric Resonator Antenna Handbook.

[4] Gao Y., Feng Z.H., Zhang L. (2011). Compact CPW-Fed dielectric resonator antenna with dual polarization. IEEE Antennas Wirel. Propag. Lett..

[5] Chair R., Kishk A.A., Lee K.F. (2006). Hook- and 3-D J-shaped probe excited dielectric resonator antenna for dual polarization applications. IEEE Proc.-Microw. Antennas Propag..

[6] Sun Y.X., Leung K.W. (2013). Dual-Band and wideband dual-polarized cylindrical dielectric resonator antennas. IEEE Antennas Wirel. Propag. Lett..

[7] Tang X.R., Zhong S.S., Kuang L.B., Sun Z. (2009). Dual-polarized dielectric resonator antenna with high isolation and low cross-polarization. IET Electron. Lett..

[8] Kowalewski J., Eisenbeis J., Jauch A., Mayer J., Kretschmann M., Zwick T. (2020). A mmW broadband dual-polarized dielectric resonator antenna based on hybrid modes. IEEE Antennas Wirel. Propag. Lett..

[9] Zou L.F., Abbott D., Fumeaux C. (2012). Omnidirectional cylindrical dielectric resonator antenna with dual polarization. IEEE Antennas Wirel. Propag. Lett..

[10] Zou L.F., Fumeaux C. (2011). A cross-shaped dielectric resonator antenna for multifunction and polarization diversity applications. IEEE Antennas Wirel. Propag. Lett..

[11] Lin T., Chiu T., Chang Y., Hsieh C., Chang D. Design of 60-GHz dual-polarization dielectric resonator antenna. Proceedings of the 2016 46th European Microwave Conference (EuMC).

[12] Ali A., Yun J., Ng H.J., Kissinger D., Giannini F., Colantonio P. Sub-THz on-chip dielectric resonator antenna with wideband performance. Proceedings of the 2019 14th European Microwave Integrated Circuits Conference (EuMIC).

[13] Deng X., Li Y., Liu C., Wu W., Xiong Y. (2015). 340 GHz on-chip 3-D Antenna with 10 dBi gain and 80% Radiation Efficiency. IEEE Trans. Terahertz Sci. Technol..

[14] Wang L., Sun W.Z. A 60-GHZ differential-fed circularly polarized on-chip antenna based on 0.18-μm COMS technology with AMC structure. Proceedings of the IET International Radar Conference 2015.

[15] Sun M., Zhang Y.P., Chua K.M., Wai L.L., Liu D., Gaucher B.P. (2008). Integration of Yagi antenna in LTCC package for differential 60-GHz radio. IEEE Trans. Antennas Propag..

[16] Li B., Leung K.W. (2008). On the differentially fed rectangular dielectric resonator antenna. IEEE Trans. Antennas Propag..

[17] Lee E., Ong M.L.C. Aperture coupled differentially fed DRAs. Proceedings of the Asia Pacific Microwave Conference.

[18] Guo S.J., Wu L.S., Leung K.W., Mao J.F. (2018). Microstrip-fed differential dielectric resonator antenna and array. IEEE Antennas Wirel. Propag. Lett..

[19] Fang X.S., Leung K.W., Lim E.H., Chen R.S. (2010). Compact differential rectangular dielectric resonator antenna. IEEE Antennas Wirel. Propag. Lett..

[20] Hao C.X., Li B., Leung K.W., Sheng X.Q. (2011). Frequency-tunable differentially fed rectangular dielectric resonator antennas. IEEE Antennas Wirel. Propag. Lett..

[21] Sun Y.X., Leung K.W., Mao J.F. (2018). Dualfunction dielectric resonator as antenna and phase-delay-line load: Designs of compact circularly polarized/differential antennas. IEEE Trans. Antennas Propag..

[22] Tong C.W., Tang H., Li J., Yang W.W., Chen J.X. (2019). Differentially coplanar-fed filtering dielectric resonator antenna for millimeter-wave applications. IEEE Antennas Wirel. Propag. Lett..

[23] Gupta R.D., Parihar M.S. (2017). Differentially fed wideband rectangular DRA with high gain using short horn. IEEE Antennas Wirel. Propag. Lett..

[24] Sun S.J., Jiao Y.C., Zhang X., Gao Y. Design of a novel differential-fed wide-beam dielectric resonator antenna. Proceedings of the 2019 International Conference on Microwave and Millimeter Wave Technology (ICMMT).

[25] Tong C.W., Tang H., Qin W., Yang W.W., Zhang X.F., Chen J.X. (2019). Differentially inserted-fed compact dual-band circularly polarized dielectric resonator antenna. IEEE Antennas Wirel. Propag. Lett..

[26] Fang X.S., Shi K.P., Sun Y.X. (2020). Design of the single-/dual-port wideband differential dielectric resonator antenna using higher-order mode. IEEE Antennas Wirel. Propag. Lett..

[27] Tang H., Chen J.X., Yang W.W., Zhou L.H., Li W.H. (2017). Differential dual-band dual-polarized dielectric resonator antenna. IEEE Trans. Antennas Propag..

[28] Tang H., Tong C.W., Chen J.X. (2018). Differential dual-polarized filtering dielectric resonator antenna. IEEE Trans. Antennas Propag..

[29] Wang X.Y., Tang S.C., Yang L.L., Chen J.X. (2020). Differential-fed dual-polarized dielectric patch antenna with gain enhancement based on higher order modes. IEEE Antennas Wirel. Propag. Lett..

[30] Sun R.Y., Han R.C. Design of dual-polarized differential feed dielectric resonator antenna. Proceedings of the 2015 IEEE MTT-S International Microwave Workshop Series on Advanced Materials and Processes for RF and THz Applications (IMWS-AMP).

[31] Chen J.X., Tang H., Li Y.L., Qin W. (2020). Differentially fed dielectric resonators. IEEE Microw. Mag..

[32] Chu L.C.Y., Guha D., Antar Y.M.M. (2009). Conformal strip-fed shaped cylindrical dielectric resonator: Improved design of a wideband wireless antenna. IEEE Antennas Wirel. Propag. Lett..

[33] Denidni T.A., Rao Q.J., Sebak A.R. (2005). Broadband L-shaped dielectric resonator antenna. IEEE Antennas Wirel. Propag. Lett..

[34] Liang X.L., Denidni T.A. (2008). H-shaped dielectric resonator antenna for wideband applications. IEEE Antennas Wirel. Propag. Lett..

[35] Liang X.L., Denidni T.A., Zhang L.N. (2009). Wideband L-shaped dielectric resonator antenna with a conformal inverted-trapezoidal patch feed. IEEE Trans. Antennas Propag..

[36] Chair R., Kishk A.A., Lee K.F. (2006). Experimental investigation for wideband perforated dielectric resonator antenna. IET Electron. Lett..

[37] Leung K.W., So K.K. (2009). Theory and experiment of the wideband two-layer hemispherical dielectric resonator antenna. IEEE Trans. Antennas Propagat..

[38] Tang Z.Y., Liu J.H., Cai Y.M., Wang J.H., Yin Y.Z. (2018). A wideband differentially fed dual-polarized stacked patch antenna with tuned slot excitations. IEEE Trans. Antennas Propag..

[39] Yang X.J., Ge L., Wang J.P., Sim C.D. (2018). A differentially driven dual-polarized high-gain stacked patch antenna. IEEE Antennas Wirel. Propag. Lett..

[40] Liu N.W., Zhu L., Zhang X., Choi W.W. (2017). A wideband differential-fed dual-polarized microstrip antenna under radiation of dual improved odd-order resonant modes. IEEE Access.

[41] Fang X.S., Leung K.W. (2012). Linear-/circular-polarization designs of dual-/wide-band cylindrical dielectric resonator antennas. IEEE Trans. Antennas Propag..

[42] Fang X.S., Chen S.M. (2019). Design of the wide dual-band rectangular souvenir dielectric resonator antenna. IEEE Access.

[43] Li W.W., Leung K.W. (2013). Omnidirectional circularly polarized dielectric resonator antenna with top-loaded alford loop for pattern diversity design. IEEE Trans. Antennas Propag..

